# “We will tell everyone!” Capturing the impact of public and patient involvement in music therapy and dementia research through songwriting

**DOI:** 10.1177/14713012251333839

**Published:** 2025-04-23

**Authors:** Lisa Kelly, Carl Corcoran, Gerry Paley, Nuala Paley, Kevin Quaid, Helena Quaid, Helen Rochford-Brennan, Carmel Geoghegan, Ita Richardson, Hilary Moss

**Affiliations:** Irish World Academy of Music and Dance, 8808 University of Limerick, Ireland; The Health Research Institute (HRI), University of Limerick, Ireland; Lero, The Science Foundation Ireland Research Centre for Software, Ireland; Irish World Academy of Music and Dance, 8808 University of Limerick, Ireland; The Alzheimer Society of Ireland, Ireland; The Health Research Institute (HRI), University of Limerick, Ireland; Lero, The Science Foundation Ireland Research Centre for Software, Ireland; Irish World Academy of Music and Dance, 8808 University of Limerick, Ireland; The Health Research Institute (HRI), University of Limerick, Ireland

**Keywords:** songwriting, dementia, music therapy, public and patient involvement, arts-based research

## Abstract

This paper describes and presents findings from a doctoral research project where songwriting was used as an approach to capture the impact of Patient and Public Involvement (PPI) in research. This paper is situated in the context of a larger research project which explored how telehealth music therapy can support people with dementia and their family caregivers living in the community. The research was guided by three PPI contributors with dementia with support from their family care partners to ensure the relevance of research outputs for people with dementia living in the community. To capture their experiences of being involved in this research, they collaboratively wrote a song entitled ‘We Will Tell Everyone’, about living well with dementia and the impact of being involved in dementia research. This paper presents the process of writing the song, the lyrics of the original song, alongside the PPI contributors experiences. Qualitative research using Interpretative Phenomenological Analysis was undertaken to analyse the process. Three themes emerged: (a) An empowering experience, (b) the importance of collaboration, respect and listening, and (c) a message of hope. This paper demonstrates how arts-based research methods such as songwriting can make research findings more impactful and offers a creative and accessible approach to capture the voices and lived experiences of people with dementia in research.

## Introduction

This paper presents reflections from a doctoral research project where song writing was used as an approach to capture the impact of Patient and Public Involvement (PPI) in music therapy and dementia research. PPI refers to “doing research with or by the public, rather than to, about, or for them” (INVOLVE, 2012). In health research, PPI is becoming increasingly popular due to the growing recognition that it has the capacity to increase the relevance and utility of research outputs to the population in question and the public, while ensuring that research deliverables can be recognised as relevant to and connected with people’s lives and well-being (Grotz et al., 2020; Poland et al., 2019). Without the involvement of PPI contributors who have experiential knowledge through their direct/lived experiences, it is easy for researchers to make assumptions or miss significant issues when making decisions about research (Staley, 2017). Thus, involving people with lived experience through PPI enhances the integrity, impact, quality and relevance of research (Miah et al., 2019).

Few studies specifically evaluate the impact of PPI in doctoral research (Coupe & Mathieson, 2020; Dawson et al., 2020; Tomlinson et al., 2019). This may be attributable to the wide diversity of context-specific PPI aims and approaches in research (Crocker et al., 2017). There is a large body of literature regarding PPI in research. However, the PPI process and impact in doctoral research projects are seldom reported in peer-reviewed papers (Coupe & Mathieson, 2020; Smith et al., 2024). This has resulted in the narrative of the research journey including researchers’ and PPI contributors’ personal reflections of involvement being excluded (Dawson et al., 2020) and guidance on how to operationalise PPI effectively (Smith et al., 2024). Time and financial constraints may also contribute to lack of confidence in incorporating PPI in doctoral research (Smith et al., 2024).

### Involvement – something to be evaluated or described?

Staley and Barron (2019) reconceptualise involvement in research as a conversation that supports two-way learning rather than as an intervention which should be evaluated. Rather it is “evolutionary, in unpredictably progressing through a series of interrelated episodes of learning, rather than following a linear, fixed path” (p. 4). These interactions often impact on research by influencing the design, delivery and the dissemination of research findings (Staley et al., 2017). When involvement is reframed as a conversation that supports learning, rather than something that should be evaluated, it highlights the reciprocal nature of the process. In essence, involvement should be tailored to the research context and the needs of the individuals involved. We agree with this commentary; involvement in research is a dynamic process, and flexibility and responsiveness to the context is key. The quality of the interactions between researchers and the public is what constitutes best practice, and the chosen method should not restrict or limit the necessary responsiveness required when conducting PPI. After all, “it is difficult to quantify learning from experience” (p. 6).

The involvement of people with a diagnosis of dementia in PPI and engagement in research design and delivery is an emerging field (Beresford-Dent et al., 2022) and has been recommended by international dementia organisations such as Alzheimer Europe (Gove et al., 2018). Guidance and resources to facilitate best practice when designing and conducting research involving people with dementia has also been published (DEEP; National Institute for Health and Care Excellence, 2021). There is a need to adopt flexible approaches rather than a one-size-fits-all model when working with PPI contributors and to consider that different approaches may be appropriate for varying contexts (Dawson et al., 2020). For PPI contributors with a diagnosis of dementia, additional support may be required to facilitate meaningful participation where difficulties with language, memory or other cognitive impairments may be experienced (Burton et al., 2019; Gove et al., 2018). Furthermore, cognitive decline associated with dementia often involves memory difficulties, perceptual abnormalities, and communication deficits (Lloyd et al., 2006). Practical strategies have been implemented by various researchers to combat some of these difficulties when those affected are involved in research, including the adaptation of reading materials, reimbursement of time and travel as well as ensuring the meetings were held in an accessible or familiar environment (Clark et al., 2020; Litherland et al., 2018; Parveen et al., 2018; Øksnebjerg et al., 2020).

This paper presents an alternative and creative approach to engaging with PPI contributors in dementia research. It introduces the concept of using songwriting as a tool to facilitate meaningful interaction and discussion with PPI contributors with dementia.

### Setting the context

The research project referred to in this paper is an exploratory phenomenological research study which explored how telehealth music therapy can support people with dementia and their family caregivers living in the community (Kelly, 2023). From the outset of the doctoral research project in 2020, the research was guided by three PPI contributors with dementia with support from their family members and care partners to ensure the relevance of research outputs for people living with dementia in the community. PPI was incorporated at each stage of the research process through embedded consultation. The PPI contributors were involved in the research planning and development phase where they developed research priorities, ensured accessibility and appropriateness of language in an online survey distributed to people with dementia and their caregivers, and aided in the recruitment of people with dementia and their supporters to other phases of this research. The project discussed in this paper can be described as the culmination of the PPI process of the overall doctoral study, which was co-produced collaboratively by the PPI contributors with assistance from the researcher (also a music therapist) and a community musician/songwriter. The music therapist recognised that songwriting skills would enhance this aspect of her work with the PPI contributors, and invited a songwriter who was also a professional community musician, to participate, after discussion with the PPI contributors. The co-production model falls under the umbrella term of public involvement and is characterised by the reciprocal nature of the relationships and collaborative process involved (Hughes & Duffy, 2018).

Over the duration of the doctoral research study, the PPI contributors shared their lived experience and experiential knowledge of dementia which informed the design and delivery of this research project. During these interactions and dialogues, the researcher and the PPI contributors became acquainted and critical friendships began to emerge. The topic of conversation organically turned to the PPI contributor’s relationship with music, knowledge of music therapy, and their reason for wanting to be involved in research. For the researcher, who is also a music therapist, this planted the seed of how she may share her experiential and professional knowledge with the PPI contributors and offer them a platform to share their voices and tell their story to a wider audience.

Thus, this paper provides reflections on the use of songwriting to demonstrate the impact of PPI in music therapy and dementia research. The aim is not to provide evidence or a ‘method’, but rather highlight that by reframing involvement as mutual conversations that support learning, and by tailoring the context and the needs of the individuals involved, involvement can be co-produced in a meaningful, creative and accessible way for people with dementia.

### Songwriting – offering a voice to people with dementia

In music therapy discourse, songwriting is gaining recognition as a supportive intervention for people with dementia and their family caregivers (Baker, 2015), offering a potent and accessible way of giving voice to people with dementia to share their unique experiences (Tamplin & Clark, 2020). This is important given that the voices of people with dementia are largely missing from the current discourse in music therapy and dementia research (Baker & Stretton-Smith, 2017). In a concerted effort to measure the impact and effectiveness of music therapy for people with dementia, many researchers have placed emphasis on designing large scale randomised controlled trials. While this approach is important to increase the validity and trustworthiness of music therapy in aged-care and community settings, purely quantitative approaches to research risk placing people with dementia as passive recipients of care rather than active participants who have the right to autonomy and agency over their care (McDermott et al., 2013).

To date, songwriting as a method has not been documented in the literature within the context of PPI and dementia research. Notwithstanding, there is an emerging body of literature on songwriting for people with dementia, including the experiences of a ten-week group therapeutic songwriting programme for people with dementia and their family caregivers have been reported (Baker & Stretton-Smith, 2017). Participants with dementia described the experience as creative, collaborative and motivating, which led to enhanced feelings of social connection, belonging, confidence, and achievement. Nonetheless, songwriting for people with dementia is sparsely reported in music therapy literature, and this may be due to an assumption that songwriting may be too cognitively demanding for this population (Clark et al., 2021). However, people with dementia are often able to access musical knowledge and skills using intact implicit or procedural musical memory (Jacobsen, 2015). Thus, with careful planning, facilitators can implement techniques to assist with overcoming cognitive challenges such as musical cues, the use of sensitive questioning, and empathetic probing to combat cognitive impairments and enable participation (Baker, 2015). Additionally, the creation of a welcoming and positive social environment can enable a person with dementia to feel empowered and comfortable enough to contribute (Brooker & Latham, 2016; Kelly et al., 2023). Contributions through creative means such as songwriting or music-making may promote personhood (Kitwood, 1997) and better capture the voices and lived experience of people with dementia in research (Clark et al., 2021). The medium of song is particularly appropriate as it provides direct access to a person’s emotional world, thoughts, attitudes, values and beliefs (Bruscia, 1998).

## Method

### Research approach

As we were interested in understanding the lived experience of the PPI contributors, Interpretative Phenomenological Analysis (IPA) was deemed the most suitable qualitative model as it takes an idiographic perspective, is concerned with representing the essence of the lived experience (Creswell, 2007), and can enhance professional knowledge (Neubauer et al., 2019). Phenomenological research operates from a constructionist epistemological position promoting discovery and description (Ghetti, 2016).

In arts-based research, theory and practice are intertwined with the aim of addressing research questions in a holistic manner (Leavy, 2020). Arts-based research practices are a set of methodological tools used by researchers during any or all stages of the research process. Adapting the principles of the creative arts, these novel research tools address research questions using music, visual art, creative writing, and other art forms to engage participants. They offer qualitative researchers a “rich, versatile means of collecting data that embody the participants’ experiences in systematic and inventive ways” (Beer, 2016, p. 33). In the context of this study, we conjectured that songwriting would enable the PPI contributors to share their experiences through creative means, and to articulate the impact of involvement in dementia research.

### The participants

Ethical approval was sought and obtained through the Faculty of Arts Humanities and Social Sciences Research Ethics Committee at The University of Limerick (2020-10-12-AHSS). The PPI contributors were recruited to this study via The Alzheimer Society of Ireland (ASI) at the commencement of this doctoral research project in Autumn 2020. A research officer was assigned by the ASI to ensure the safety of the PPI contributors. Informed consent was obtained in both written and verbal format prior to the commencement of the research. The PPI contributors recruited to this project were three people with varying diagnoses of dementia (Alzheimer’s disease, early onset, Lewy body dementia) who are living in the community (Gerry, Helen & Kevin) who were supported at in person events by their family members and supporters (Nuala, Carmel & Helena). The six PPI contributors formally agreed to be involved as co-authors in the project outputs. This article was primarily drafted by Lisa (doctoral researcher) and circulated to the PPI contributors who were invited to contribute or make edits and/or suggestions.

### The songwriting workshops

The songwriting workshops were carried out in two separate phases. The first phase took place in August 2022 and was funded by PPI Ignite @UL. The second phase took place in March 2023 and was funded by a creative dissemination bursary awarded to the primary doctoral researcher by the ASI to complete and professionally record the song.

On both occasions, the songwriting workshop was facilitated by Carl Corcoran, an esteemed songwriter and community musician and co-facilitated by Lisa, the primary doctoral researcher who is also a music therapist, at the Irish World Academy of Music and Dance, University of Limerick. Available instruments included a baby grand piano, a guitar, and some small percussion instruments. Principles of confidentiality, a safe space and humanistic principles of unconditional positive regard and empathy were protected by the music therapist and the songwriter brought expertise in facilitating community groups in songwriting as well as being a successful songwriter. A whiteboard was used to write down any lyrics or ideas which the PPI contributors put forth to the group. Once these lyrics were refined and shaped to the template of the song, they were transcribed to a Microsoft Word document which was made visible in large text to the PPI contributors via screen share using a HDMI cable. A hard and digital copy of the lyrics were distributed to the members of the group once completed.

The lyrics of the song were solely generated by the PPI contributors and their supporters. This was important given that the voices of people with dementia are largely missing in dementia research. These lyrics emerged organically from a conversation in the first songwriting workshop. Phrases and words which resonated with their experience were written on a whiteboard, and later reconstructed to convey the message they wanted to share. Carl, who has extensive experience as a songwriter and community musician, scaffolded these lyrics melodically and suggested a chordal structure and musical arrangement in consultation with the members of the group. The final output was entitled ‘We Will Tell Everyone!’. The lyrics of the song are presented below. The PPI contributors also gave a public performance to a live audience at The Irish World Academy of Music and Dance as part of the Tower Seminar series. The performance evoked a strong emotional response from the audience and received a standing ovation. This performance prompted additional funding to be allocated to make a professional audio and video recording of the song to be released to the general public. The final output of the project is linked here.

The Song: ‘We Will Tell Everyone!’

Verse 1

A photo captures a moment in time and a lifetime of memories.

It helps me remember thoughts forgotten, reflection of what used to be.

We’ve developed a negative of black and white turning our life into colour.

Filled with joy and happiness, a life that is a whole lot fuller.

Chorus

Who will we tell? We will tell everyone!

I’ll continue to be me. Still telling everyone!

We’ll shout it from the rooftops, the mountains and the hills

Living life to our fullest, we do it still.

Who will we tell? We will tell everyone!

Verse 2

With families and friends by our side we’ve been able to make new connections

Our memories may fade but emotion stays, it helps us with our recollections.

With new friends and old, we have struck gold, our new journey has begun.

We will never let go, we need everyone to know, our hearts will forever stay young

Chorus

Who will we tell? We will tell everyone!

I’ll continue to be me. Still telling everyone!

We’ll shout it from the rooftops, the mountains and the hills

Living life to our fullest, we do it still.

Who will we tell? We will tell everyone!

Bridge

We still dance, we still sing, we still laugh – do everything.

We still work, we still drive – We are alive!

Repeat Chorus

### Data collection and analysis

At the end of the second workshop, participants were invited to share their experience of the songwriting workshops. This discussion was led by Lisa. No guiding questions were posed, and the PPI contributors were encouraged to openly discuss their experience within the group setting. The discussion was audio recorded on a laptop and transcribed to a password protected Microsoft word document. The audio recording was subsequently deleted.

Data were analysed using the flexible guidelines of IPA (Smith et al., 2009). Analysis in IPA always involves interpretation. It is stated that IPA researchers are engaged in a double hermeneutic. This is because the researcher endeavours to make sense of the participant who is also attempting to make sense of their experience. Thus, the end result is a tentative and subjective account of how the researcher “thinks the participant is thinking” (p. 80). Intersubjective framing navigates the complexities of individual perspectives while fostering a shared understanding essential for effective collaboration. Despite the subjectivity, this method is also rigorous and systematic in its application. IPA also recognises that access to experience is dependent on what the participant tells us about that experience (Smith et al., 2009). Firstly, the transcripts were read and re-read and sections which were deemed significant were colour coded and any comments were placed in the margin of the document. By engaging in an interpretative relationship with the highlighted transcripts, initial notes and emerging codes, a structure emerged which reflected the experiences of the PPI contributors. The final interpretations of the data were returned to the PPI contributors to ensure the researcher has recorded and represented their responses accurately (Birt et al., 2016).

## Findings

Three themes emerged from the data analysis. This included (a) an empowering experience, (b) the importance of collaboration, respect and listening and (c) a message of hope.

### An empowering experience

The PPI contributors and their supporters spoke of how the songwriting process made them feel empowered. Helen, who was initially emotional when she heard the lyrics in the bridge section, said: “To be able to tell the world that you can still dance, you can drive, you can go on enjoying your life … it’s powerful”. She described how she was a “nervous wreck” when she entered the room and “didn’t know what to say”. She articulated that she was surprised when the conversation started, and we began to generate lyrics immediately, commenting that “your life just flows out”. Kevin spoke of how the experience brought him out of his comfort zone:

I just realised today that there’s nothing we can’t do… Personally, I can’t thank the two of you enough and the [others] for bringing me out of my comfort zone, or what I thought was my comfort zone. I’ve now realised that nothing is impossible especially when you have a diagnosis. It’s then when things become really possible.

Nuala described herself and Gerry’s experiences of writing the song as “one of the main things that has affected us and helped us and brought us forward in our journey”. She later said, “words don’t even fill what this means to all of us. It really, really is very deep, very empowering, and a very strong, sincere message to others”.

### The importance of collaboration, respect and listening

A collaborative process and supportive and respectful environment were deemed essential when writing the song collectively. Whilst these may seem to be separate elements, the analysis revealed that collaboration only happens successfully when there is respect and listening between parties and that these are essential elements that must sit together in any successful participatory project. Kevin described the process as unique and commented that “no one took ownership, or leadership, or nothing. The amount of times that I heard today ‘that’s a brilliant idea’. No one said. ‘No, no, that won’t work’.” From Gerry’s experience the group worked well together because “we all participate, we all contribute, and we are there for each other”. For Helen, the facilitators listening to, and incorporating her contributions was important:

You’ve listened to our words right through to today to make changes and that …You’ve listened. Its only that one word – listening. You’ve listened to us, and you’ve changed it, and you’ve changed it up to the last minute, which is great because you made us, well you’ve certainly made me feel extremely comfortable. I couldn’t say that when I walked through the door that I would be that comfortable. But I’m extremely comfortable today. Emotional but comfortable. It’s just… you’ve listened.

For Carmel, respect was pivotal to the collaborative process: “It’s probably one of the best pieces that I’ve been involved in as regards respect…feeling respected and equal, because that does not always happen”.

### A message of hope

Helen described how the song gave her a sense of hope for the future, saying “there is no medicine for us. So, what we have left is hope. You brought a lot of hope into my life”. For Helena, the song reminded her of when her husband Kevin was diagnosed with Lewy Body dementia and how difficult that was. She described the experience as more than a ‘research project’. And how the process made her feel uplifted and positive:

I was very emotional because I found the whole experience really lifted us up to a new level … It was beautiful and I could think back on the day of Kevin’s Lewy body dementia diagnosis. That day when we left, we were given no guidelines in how to live well with dementia…and we were told it was a progressive disease. But today, it’s progressively going forward with happiness.

Helena later commented on the positive message that the song conveys about living well with dementia: “I feel that Gerry, Helen, and Kevin all brought their openness and honesty into the conversation, and into the lyrics. Living well with dementia is what it’s all about”. For Nuala, the key message in the song is that there is life after a dementia diagnosis and recalled that “it’s a surprise in the beginning. You know, that there is something else”. Gerry echoed this:

When I was diagnosed with dementia, I thought to myself, what’s the next step for me? What’s going to happen to me? Will I be able to function, or will I be able to still carry on? Thankfully with the help of medication and that, I’m still able to do everything that I want to do…I can still live a good life and function and enjoy life.

## Discussion

This paper highlights how songwriting was used to capture the impact of public and patient involvement in music therapy and dementia research. Moreover, it dually highlights the experiences of PPI contributors being involved in research. These dual perspectives form the basis for this discussion.

As a qualitative research method, arts-based research can contribute to illuminating and exploring the lived experience of dementia (Moss & O’Neill, 2019). The arts act as a “reflective tool for learning and understanding a complex health condition, as well as creating opportunities for increased understanding and public awareness of dementia” (p. 2008). For the PPI contributors with a diagnosis of dementia in this study, the original song was not only reflective of their experiences of being involved in research, but also aimed to challenge the stigma associated with a dementia diagnosis and to promote awareness of the disease. Essentially, they wanted to reconstruct the narrative from a negative experience to a positive reflection of how to live well with dementia, thus challenging the stigma regarding the creativity of people with dementia. This was demonstrated in the impact of the song at several performances, where audience members and performers noted that some of the song lyrics changed their perception, whilst the participants described it as ‘an anthem’ telling the world that people with dementia can still drive, work, dance and are alive! Indirectly, this study aligned with the process of therapeutic songwriting, a process which encourages participants to explore their thoughts, feelings, attitudes, and memories and guides them to translate ideas into lyrics and the creation of a song (Baker, 2015; Clark et al., 2024).

The facilitation style was also pivotal to the effective delivery of the songwriting workshop and achieving a sense of empowerment. The creation of an encouraging positive social environment was key to enable the PPI contributors to share their personal experiences. Rodgers (1993) described external conditions that nurture and foster the internal conditions necessary to promote creativity: Psychological safety, psychological freedom, and offering stimulating and challenging experiences. The provision of an environment where external evaluation is absent contributes to finding “a space psychological space, freeing the person to be who they are and knowing there are no winners or losers, and each person’s contribution is genuinely valued” (Lee & Adams, 2011, p. 8). Enabling factors to guide authentic partnerships when collaborating with people with dementia have also been identified (Dupuis et al., 2016). These include working with a diverse range of individuals and understanding and supporting their personal strengths and resources, providing a safe space where group members can share their experiences openly, valuing and including diverse perspectives, ensuring open communication, and regular feedback and dialogue. In the context of person-centred dementia care, Kitwood (1997) describes facilitation as enabling a person with dementia to do what otherwise they would not be able to do, thus resulting in a collaborative interaction. The true meaning of collaboration is ‘working together’ and this was highlighted as extremely important by the PPI contributors. Within the context of this study, is important to note the skill and nuance brought to this process by an experienced music therapist and community musician who provided space and listened carefully to the contributors’ responses. We recommend further research to interrogate what constitutes an effective and excellent facilitation style when designing and providing music interventions/workshops for this cohort (Moss et al., 2021).

While the specific focus of this paper is demonstrating the impact of PPI involvement in research through creative means, this approach may also be adapted as a creative dissemination method. Evidence supports involving PPI contributors in the dissemination of research findings, as they can translate findings into meaningful, everyday messages and demonstrate relevance to the public (Tomlinson et al., 2019). The inclusion of arts-based approaches in research can make research findings more impactful to the wider audience. As articulated by Beer (2016), when creative arts are included in the dissemination of research findings, “otherwise inaccessible data pieces become part of an interactive event for readers in which they not only intellectually comprehend how a participant experienced the phenomenon being studied, but also emotionally and intuitively respond to what they hear” (p. 33). Additionally, the artistic representation of ideas and experiences through the arts can make research findings more accessible to a wider audience (Knowles & Cole, 2008), make lasting impressions, and “evoke emotions, promote reflection, and transform the way people think” (Leavy, 2020, p. 305). This was clearly evident by the emotional response which this song evoked in a live audience. Arts-based research methods also present opportunities to pose new questions, gain a deeper understanding, and enhance knowledge transition through creative and evocative modes of communicating evidence (Boydell et al., 2016).

### Limitations and recommendations

It must be acknowledged that Lisa had a complex dual role as both a researcher and music therapy facilitator in this research project. The research involved reflecting upon personal experiences of facilitating the sessions, and theorising and contextualising this experience. It is possible that the PPI contributors could have been less likely to share negative experiences in the discussion because of this dual role. The inclusion of an external facilitator to lead the discussion was considered. However, given the confusion people with dementia may experience, the familiarity of the researchers face may have contributed to feelings of safety and potentially supported participants as they described their experiences (Baker & Stretton-Smith, 2018). Thus, this dual role was embraced and carefully monitored through peer and research supervision, repeated analysis of the recorded transcript and the implementation of member checking. Intersubjectivity was promoted through member checking, with the aim of exploring the credibility of results and reducing research bias. This technique involved returning the data to the PPI contributors to check for accuracy and to ensure the researcher has recorded and represented their responses appropriately (Birt et al., 2016). To mitigate this possible subjectivity and bias, we would recommend that the facilitation of the intervention and data collection and analysis be completed independently of one another by separate researchers if this study were replicated in the future.

## Conclusion

In this paper, we have highlighted how representation, narrative, and empowerment can be achieved through music, and specifically songwriting in arts-based research (Daykin, 2004). The experiences presented in this paper act as a first step in exploring how songwriting, and perhaps arts-based research more broadly, can be used to document the impact of PPI in dementia research and potentially be used as a creative dissemination method. The use of creative methods such as songwriting can help researchers access, highlight, describe, and explain that which is often inaccessible by other more traditional research practices (interviews, focus groups, outcome measures) when working with people with dementia. More importantly, it offers people with dementia a creative and potent means to express their voice and share their story. For the PPI contributors of this music therapy and dementia research project, songwriting was an empowering and collaborative process that represents their lived experience and gave them a sense of hope.

## Data Availability

The datasets used and/or analysed during the current study are available from the corresponding author on reasonable request.*

## References

[bibr1-14713012251333839] Baker F. A. (2015). Therapeutic songwriting: Developments in theory, methods, and practice. Palgrave Macmillan.

[bibr2-14713012251333839] Baker F. A. Stretton-Smith P. A. (2018). Group therapeutic songwriting and dementia: Exploring the perspectives of participants through interpretative phenomenological analysis. Music Therapy Perspectives, 36(1), 50–66. DOI: 10.1093/mtp/mix016.

[bibr3-14713012251333839] Beer L. E. (2016). From embedded to embodied: Including music in arts-based music therapy research. Music Therapy Perspectives, 34(1), 33–40. DOI: 10.1093/mtp/miv006.

[bibr49-14713012251333839] Beresford-Dent J. Sprange K. Mountain G. Mason C. Wright J. Craig C. Birt L. (2022). Embedding patient and public involvement in dementia research: Reflections from experiences during the “Journeying through Dementia” randomised controlled trial. Dementia (London), 21(6), 1987–2003. DOI:10.1177/14713012221106816 35670381 PMC12064857

[bibr4-14713012251333839] Birt L. Scott S. Cavers D. Campbell C. Walter F. (2016). Member checking: A tool to enhance trustworthiness or merely a nod to validation? Qualitative Health Research, 26(13), 1802–1811. DOI: 10.1177/1049732316654870. 27340178

[bibr5-14713012251333839] Boydell K. M. Hodgins M. Gladstone B. M. Stasiulis E. Belliveau G. Cheu H. Kontos P. Parsons J. (2016). Arts-based health research and academic legitimacy: Transcending hegemonic conventions. Qualitative Research, 16(6), 681–700. DOI: 10.1177/1468794116630040.

[bibr6-14713012251333839] Brooker D. Latham I. (2016). Person-centred dementia care: Making services better with the VIPS framework (2nd ed.). Jessica Kingsley Publishers.

[bibr7-14713012251333839] Bruscia K. (1998). The dynamics of music psychotherapy. Barcelona Publishers.

[bibr8-14713012251333839] Burton A. Ogden M. Cooper C. (2019). Planning and enabling meaningful patient and public involvement in dementia research. Current Opinion in Psychiatry, 32(6), 557–562. DOI: 10.1097/YCO.0000000000000548. 31306247

[bibr9-14713012251333839] Clark I. Christopher N. Stretton-Smith P. Lawson K. (2024). The experiences of people living with dementia and their care partners participating in an online therapeutic songwriting program. Dementia, 23(2), 251–271. DOI: 10.1177/14713012231224069. 38131325 PMC10807188

[bibr10-14713012251333839] Clark I. N. Baker F. A. Tamplin J. Lee Y. C. Cotton A. Stretton-Smith P. A. (2021). “Doing things together is what it’s about”: An interpretative phenomenological analysis of the experience of group therapeutic songwriting from the perspectives of people with dementia and their family caregivers. Frontiers in Psychology, 12, 598979. DOI:10.3389/fpsyg.2021.598979 33868077 PMC8044441

[bibr11-14713012251333839] Clark I. N. Stretton-Smith P. A. Baker F. A. Lee Y.-E. C. Tamplin J. (2020). “It’s feasible to write a song”: A feasibility study examining group therapeutic songwriting for people living with dementia and their family caregivers. Frontiers in Psychology, 11, 1951. DOI:10.3389/fpsyg.2020.01951 32849143 PMC7426520

[bibr12-14713012251333839] Coupe N. Mathieson A. (2020). Patient and public involvement in doctoral research: Impact, resources and recommendations. Health Expectations: An International Journal of Public Participation in Health Care and Health Policy, 23(1), 125–136. DOI: 10.1111/hex.12976. 31613049 PMC6978853

[bibr13-14713012251333839] Creswell J. W. (2007). Qualitative inquiry and research design (2nd ed.). Sage Publications.

[bibr14-14713012251333839] Crocker J. C. Boylan A. M. Bostock J. Locock L. (2017). Is it worth it? Patient and public views on the impact of their involvement in health research and its assessment: A UK-based qualitative interview study. Health Expectations: An International Journal of Public Participation in Health Care and Health Policy, 20(3), 519–528. DOI: 10.1111/hex.12479. 27338242 PMC5433537

[bibr15-14713012251333839] Dawson S. Ruddock A. Parmar V. Morris R. Cheraghi-Sohi S. Giles S. Campbell S. (2020). Patient and public involvement in doctoral research: Reflections and experiences of the PPI contributors and researcher. Research Involvement and Engagement, 6(1), 23. DOI: 10.1186/s40900-020-00201-w. 32426162 PMC7216324

[bibr16-14713012251333839] Daykin N. (2004). The role of music in an arts-based qualitative inquiry. International Journal of Qualitative Methods, 3(2), 36–44. DOI: 10.1177/160940690400300203.

[bibr17-14713012251333839] DEEP . Dementia engagement and empowerment project (DEEP) the UK network of dementia voices - DEEP guides for organisations and communities. https://www.dementiavoices.org.uk/deep-resources/.

[bibr18-14713012251333839] Dupuis S. Mcainey C. A. Fortune D. Ploeg J. Witt L. D. (2016). Theoretical foundations guiding culture change: The work of the partnerships in dementia care alliance. Dementia, 15(1), 85–105. DOI: 10.1177/1471301213518935. 24419355 PMC4674759

[bibr19-14713012251333839] Ghetti C. M. (2016). Phenomenological research in music therapy. In Edwards J. (Ed.), The oxford handbook of music therapy (pp. 767–800). Oxford University Press. DOI: 10.1093/oxfordhb/9780199639755.013.15.

[bibr20-14713012251333839] Gove D. Diaz-Ponce A. Georges J. Moniz-Cook E. Mountain G. Chattat R. Øksnebjerg L. The European Working Group of People With Dementia . (2018). Alzheimer Europe’s position on involving people with dementia in research through PPI (patient and public involvement). Aging & Mental Health, 22(6), 723–729. DOI: 10.1080/13607863.2017.1317334. 28513210

[bibr21-14713012251333839] Grotz J. Ledgard M. Poland F. (2020). Patient and public involvement in health and social care research: An introduction to theory and practice. Palgrave Macmillian. DOI: 10.1007/978-3-030-55289-3.

[bibr22-14713012251333839] Hughes M. Duffy C. (2018). Public involvement in health and social sciences research: A concept analysis. Health Expectations: An International Journal of Public Participation in Health Care and Health Policy, 21(6), 1183–1190. DOI: 10.1111/hex.12825. 30159960 PMC6250854

[bibr23-14713012251333839] INVOLVE . (2012). Briefing notes for researchers: Involving the public in NHS, public health and social care research. INVOLVE. https://www.nihr.ac.uk/documents/about-us/our-contribution-to-research/how-we-involve-patients-carers-and-the-public/Going-the-Extra-Mile.pdf

[bibr24-14713012251333839] Jacobsen J.-H. Stelzer J. Fritz T. H. Chételat G. La Joie R. Turner R. (2015). Why musical memory can be preserved in advanced Alzheimer’s disease. Brain: A Journal of Neurology, 138(8), 2438–2450. DOI: 10.1093/brain/awv135. 26041611

[bibr25-14713012251333839] Kelly L. (2023). Can telehealth music therapy support people with dementia and their family caregivers living in the community? An interdisciplinary exploration involving experts by experience. Doctoral Dissertation. University of Limerick. DOI: 10.34961/researchrepository-ul.24081513.v1.

[bibr26-14713012251333839] Kelly L. Ahessy B. Richardson I. Moss H. (2023). Aligning Kitwood’s model of person-centered dementia care with music therapy practice. Music Therapy Perspectives, 41(2), 198–206. DOI: 10.1093/mtp/miad015.

[bibr27-14713012251333839] Kitwood T. (1997). Dementia reconsidered: The person comes first. Open University Press.

[bibr28-14713012251333839] Knowles J. G. Cole A. L. (Eds.), (2008). Handbook of the arts in qualitative research: Perspectives, methodologies, examples, and issues. Sage.

[bibr30-14713012251333839] Leavy P. (2020). Method meets art: Arts-based research practice (3rd ed.). The Guildford Press.

[bibr31-14713012251333839] Lee H. Adams T. (2011). Creative approaches in dementia care. Palgrave Macmillan.

[bibr32-14713012251333839] Litherland R. Burton J. Cheeseman M. Campbell D. Hawkins M. Hawkins T. Oliver K. Scott D. Ward J. Nelis S. M. Quinn C. Victor C. Clare L. (2018). Reflections on PPI from the ‘Action on living well: Asking you’ advisory network of people with dementia and carers as part of the IDEAL study. Dementia, 17(8), 1035–1044. DOI: 10.1177/1471301218789309. 30373457

[bibr50-14713012251333839] Lloyd V. Gatherer A. Kalsy S. (2006). Conducting qualitative interview research with people with expressive language difficulties. Qual Health Res, 16(10), 1386–1404. DOI:10.1177/1049732306293846 17079800

[bibr33-14713012251333839] McDermott O. Crellin N. Ridder H. M. Orrell M. (2013). Music therapy in dementia: A narrative synthesis systematic review. International Journal of Geriatric Psychiatry, 28(8), 781–794. DOI: 10.1002/gps.3895. 23080214

[bibr34-14713012251333839] Miah J. Dawes P. Edwards S. Leroi I. Starling B. Parsons S. (2019). Patient and public involvement in dementia research in the European union: A scoping review. BMC Geriatrics, 19(1), 220. DOI: 10.1186/s12877-019-1217-9. 31412788 PMC6694462

[bibr35-14713012251333839] Moss H. Lee S. Clifford A. M. Ní Bhriain O. O’Neill D. (2021). Together in song: Designing a singing for health group intervention for older people living in the community. Nordic Journal of Music Therapy, 31(5), 413–430. DOI: 10.1080/08098131.2021.2004613.

[bibr36-14713012251333839] Moss H. O’Neill D. (2019). Narratives of health and illness: Arts-based research capturing the lived experience of dementia. Dementia, 18(6), 2008–2017. DOI: 10.1177/1471301217736163. 29022362

[bibr37-14713012251333839] National Institute for Health and Care Excellence . (2021). NIHR INVOLVE. https://www.invo.org.uk/about-involve/

[bibr38-14713012251333839] Neubauer B. E. Witkop C. T. Varpio L. (2019). How phenomenology can help us learn from the experiences of others. Perspectives on Medical Education, 8(2), 90–97. DOI: 10.1007/s40037-019-0509-2. 30953335 PMC6468135

[bibr39-14713012251333839] Øksnebjerg L. Diaz-Ponce A. Gove D. Moniz-Cook E. Mountain G. Chattat R. Woods B. (2018). Towards capturing meaningful outcomes for people with dementia in psychosocial intervention research: A pan-European consultation. Health Expectations: An International Journal of Public Participation in Health Care and Health Policy, 21(6), 1056–1065. DOI: 10.1111/hex.12799. 29920881 PMC6250864

[bibr40-14713012251333839] Parveen S. Barker S. Kaur R. Kerry F. Mitchell W. Happs A. Fry G. Morrison V. Fortinsky R. Oyebode J. R. (2018). Involving minority ethnic communities and diverse experts by experience in dementia research: The caregiving HOPE study. Dementia, 17(8), 990–1000. DOI: 10.1177/1471301218789558. 30373461

[bibr41-14713012251333839] Poland F. Charlesworth G. Leung P. Birt L. (2019). Embedding patient and public involvement: Managing tacit and explicit expectations. Health Expectations: An International Journal of Public Participation in Health Care and Health Policy, 22(6), 1231–1239. DOI: 10.1111/hex.12952. 31538704 PMC6882252

[bibr42-14713012251333839] Rogers N. (1993). The creative connection: Expressive arts in healing. Science and Behaviour Books Inc.

[bibr43-14713012251333839] Smith C. Baillie J. Gill P. (2024). Importance of patient and public involvement in doctoral research involving people living with dementia. Nurse Researcher, 32(2), 39–45. DOI: 10.7748/nr.2024.e1919. 38532623

[bibr44-14713012251333839] Smith J. A. Larkin M. H. Flowers P. (2009). Interpretative phenomenological analysis: Theory, method and research. Sage.

[bibr45-14713012251333839] Staley K. (2017). Changing what researchers “think and do”: Is this how involvement impacts on research? Research: Ideas for Today’s Investors, 1(1), 158–167. DOI: 10.18546/RFA.01.1.13.

[bibr46-14713012251333839] Staley K. Barron D. (2019). Learning as an outcome of involvement in research: What are the implications for practice, reporting and evaluation? Research Involvement and Engagement, 14(5), 14. DOI: 10.1186/s40900-019-0147-1. PMC6416961 30915234

[bibr47-14713012251333839] Tamplin J. Clark I. N. (2020). Therapeutic music interventions to support people with dementia living at home with their family caregivers. In Baird A. Garrido S. Tamplin J. (Eds.), Music and dementia: From cognition to therapy (pp. 269–287). Oxford University Press.

[bibr48-14713012251333839] Tomlinson J. Medlinskiene K. Cheong V. L. Khan S. Fylan B. (2019). Patient and public involvement in designing and conducting doctoral research: The whys and the hows. Research Involvement and Engagement, 5(23), 23. DOI:10.1186/s40900-019-0155-1 31428458 PMC6697942

